# First filter feeding in the Early Triassic: cranial morphological convergence between *Hupehsuchus* and baleen whales

**DOI:** 10.1186/s12862-023-02143-9

**Published:** 2023-08-08

**Authors:** Zi-Chen Fang, Jiang-Li Li, Chun-Bo Yan, Ya-Rui Zou, Li Tian, Bi Zhao, Michael J. Benton, Long Cheng, Xu-Long Lai

**Affiliations:** 1 School of Earth Sciences, China University of Geosciences, Wuhan, 430074 P. R. China; 2 Hubei Key Laboratory of Paleontology and Geological Environment Evolution, Wuhan Center of China Geological Survey, Wuhan, 430205 P. R. China; 3 Hubei Institute of Geosciences, Hubei Geological Bureau, Wuhan, 430034 P. R. China; 4 State Key Laboratory of Biogeology and Environmental Geology, China University of Geosciences, Wuhan, 430078 P. R. China; 5 School of Earth Sciences, University of Bristol, Life Sciences Building, Tyndall Avenue, Bristol, BS8 1TQ UK

**Keywords:** Nanzhang-Yuan’an Fauna, Marine reptiles, Ichthyosauromorph, Mysticeti, Mesozoic

## Abstract

Modern baleen whales are unique as large-sized filter feeders, but their roles were replicated much earlier by diverse marine reptiles of the Mesozoic. Here, we investigate convergence in skull morphology between modern baleen whales and one of the earliest marine reptiles, the basal ichthyosauromorph *Hupehsuchus nanchangensis*, from the Early Triassic, a time of rapid recovery of life following profound mass extinction. Two new specimens reveal the skull morphology especially in dorsal view. The snout of *Hupehsuchus* is highly convergent with modern baleen whales, as shown in a morphometric analysis including 130 modern aquatic amniotes. Convergences in the snout include the unfused upper jaw, specialized intermediate space in the divided premaxilla and grooves around the labial margin. *Hupehsuchus* had enlarged its buccal cavity to enable efficient filter feeding and probably used soft tissues like baleen to expel the water from the oral cavity. Coordinated with the rigid trunk and pachyostotic ribs suggests low speeds of aquatic locomotion, *Hupehsuchus* probably employed continuous ram filter feeding as in extant bowhead and right whales. The Early Triassic palaeoenvironment of a restrictive lagoon with low productivity drove *Hupehsuchus* to feed on zooplankton, which facilitated ecosystem recovery in the Nanzhang-Yuan’an Fauna at the beginning of the Mesozoic.

**Supplementary Information:**

The online version contains supplementary material available at 10.1186/s12862-023-02143-9.

## Background

Secondarily aquatic tetrapods such as reptiles and mammals provide textbook examples of convergent evolution in feeding and locomotion [1, 2]. Most such convergences are seen in carnivorous, hunting modes of life where Mesozoic marine reptiles, whales or pinnipeds have become top predators in their ecosystems [3]. Less familiar are examples of massive filter feeders, the role taken today by numerous species of baleen whales and explored by giant Late Jurassic pachycormiform fishes [4]. In filter feeding, the baleen whales use baleen plates, loosely articulated rostral bones, large mouths and expandable throats [5], while the pachycormiform fishes evolved complex gill-arch and edentulous enlarged mouths: all these filter-adaptations aim to retain small prey items within the oral cavity [6]. It had been suggested, however, that marine reptiles could not be filter feeders (suspension feeders) because they lack the key features of fishes and mammals that enable them to feed in this way, such as gill slits of fishes or baleen of whales [7]. However, filter feeding has already been suggested in the Late Cretaceous plesiosaur *Morturneria* [8] and the Late Triassic nothosauroid *Paludidraco* [9], both based on the configuration of their dentitions and oral cavity. Further, some marine reptiles, despite not being regarded as filter feeders, used filtration while processing the food, such as *Atopodentatus unicus* and *Henodus chelyops* in the Triassic [10, 11].

Here we present compelling evidence for filter feeding in one of the earliest marine reptiles of the Mesozoic, *Hupehsuchus nanchangensis*, named by Young and Dong in 1972 [12] from the Nanzhang-Yuan’an Fauna (NYF) of southern China [13, 14]. The NYF is dated as Early Triassic (late Olenekian, Spathian, 249.2–247.2 Ma) and it includes other, but rarer, ichthyosauriforms, eosauropterygians and saurosphargiforms [14–17], but no fossils of fishes or invertebrate macrofossils [18]. In the NYF, hupehsuchians are the most diverse taxa, with five genera (*Hupehsuchus*, *Nanchangosaurus*, *Parahupehsuchus*, *Eohupehsuchus*, and *Eretmorhipis*) [12, 18–23]. The NYF fossil community differs from that of the coeval Chaohu Fauna, in which large populations of invertebrates and fishes served as food resources for marine reptiles [24].

The feeding strategy of *Hupehsuchus* has been controversial because its skull was poorly preserved. *Hupehsuchus* was first suggested as a filter-feeder by Carroll and Dong [25] based on its edentulous snout, but this hypothesis was rejected by Collin and Janis [7] because of its small narrow skull and relatively long neck. Motani et al. [26] studied the palate and mandible from the only specimen which preserved the skull in ventral view and suggested *Hupehsuchus* was a filter-feeding animal, comparable with pelicans and rorquals. These authors considered that the long neck and slender skull would not prevent filter feeding by *Hupehsuchus* [26].

**Fig. 1:**
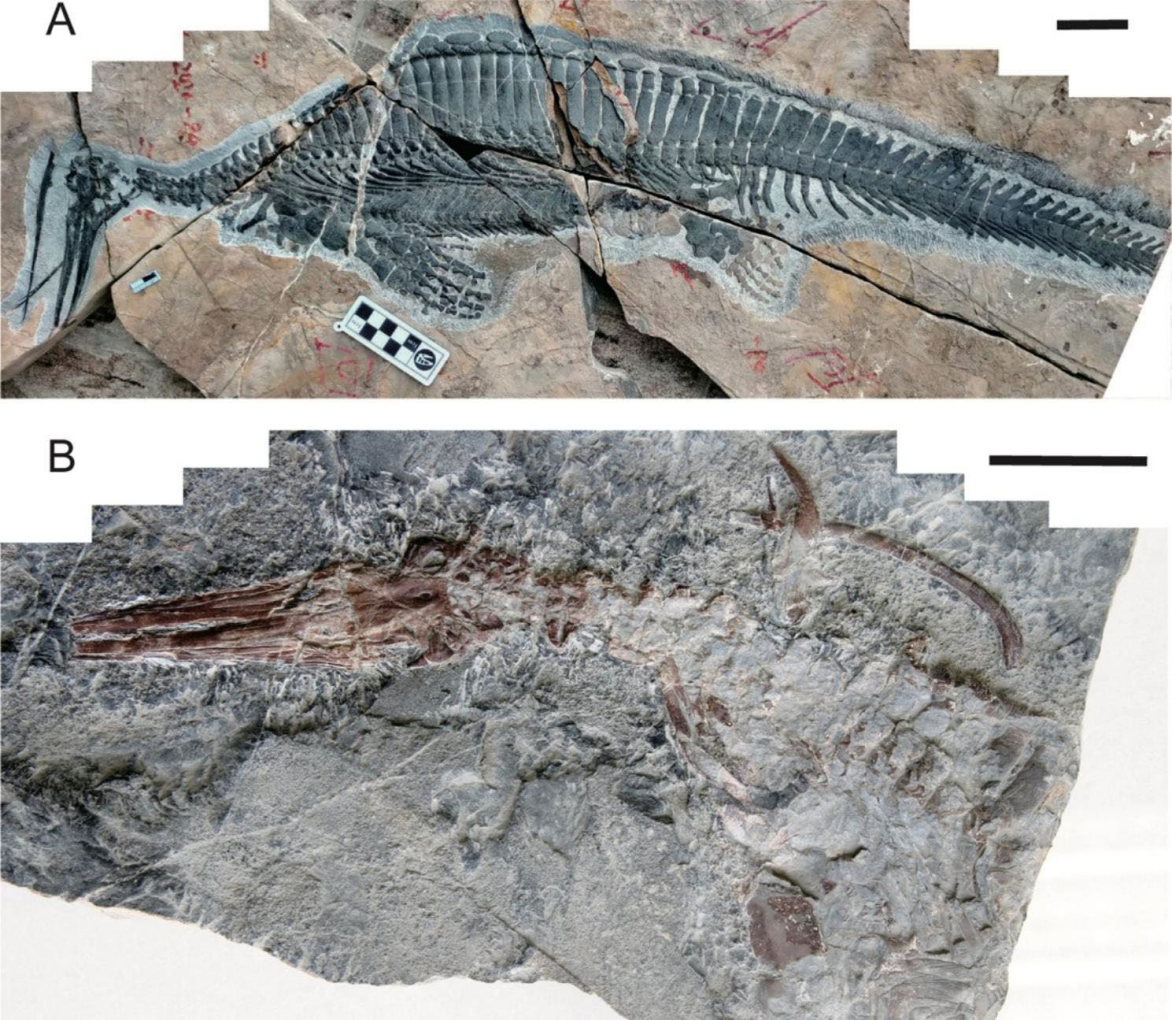
New specimens of *Hupehsuchus nanchangensis* **(A)** Photograph of 2020-NYF-84-4. **(B)** Photograph of WGSC V26007. Scale bar = 5 cm

In order to resolve this question, new skull specimens are required, especially examples that show the dorsal view of the snout. Here we report two specimens of *Hupehsuchus nanchangensis* that preserve the skull in dorsal view (Fig. 1), revealing that its cranial morphology is convergent with modern baleen whales.

## Materials and methods

### Specimens

The two new specimens of *Hupehsuchus nanchangensis*, WGSC V26007 and 2020-NYF-84-4, are respectively housed at Wuhan Centre of China Geological Survey (WGSC) and Yuan’an Geology Museum (YGM). Both specimens were collected from the Lower Triassic Member II of the Jialingjiang Formation (Lower Triassic) in Nanzhang and Yuan’an County, Hubei Province. The skeleton of WGSC 26,007 is preserved from the skull to the clavicle region, and 2020-NYF-84-4 is a nearly complete skeleton (Fig. 1). The referred specimen WGSC V26000 was studied by Motani et al. [26] and identified as *Hupehsuchus* sp., distinguished from *H. nanchangensis* by a few minor differences in vertebral count, phalangeal formula, and longitudinal spacing of limb elements.

### Geometric morphometrics and statistics

**Fig. 2:**
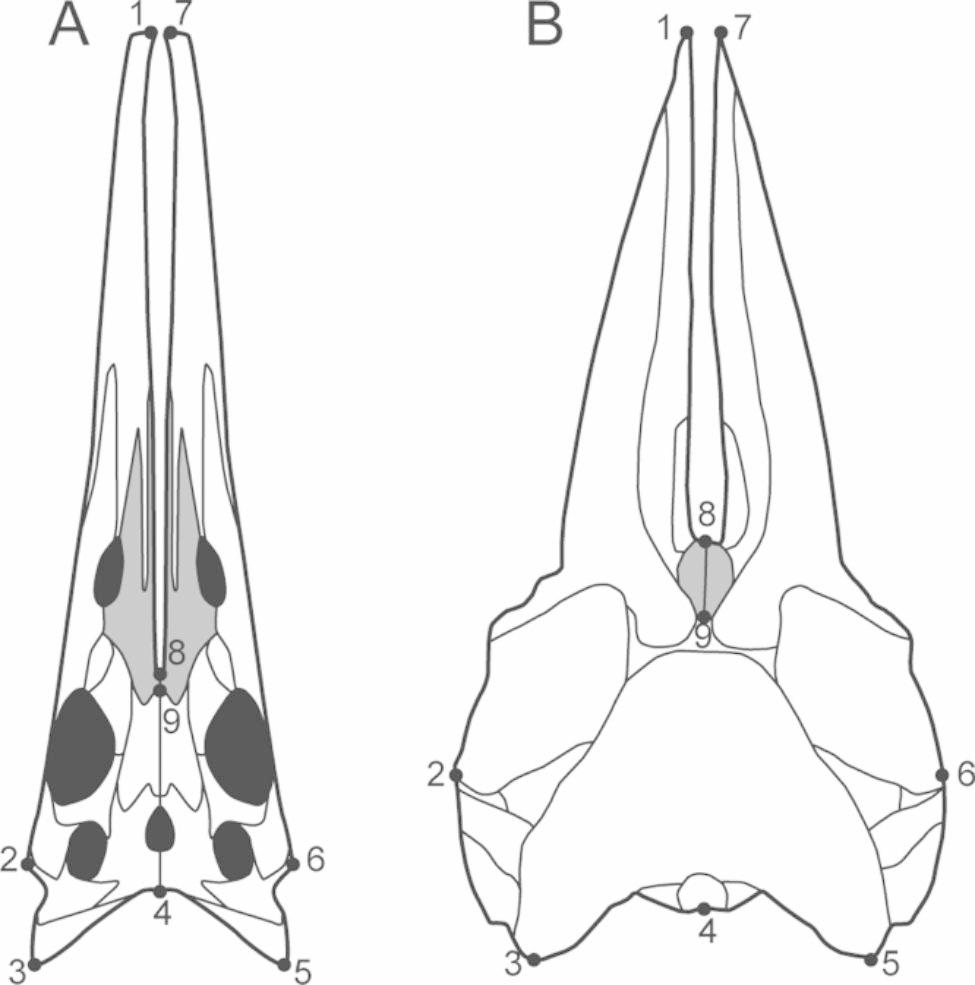
Comparison of the skull roof of *Hupehsuchus nanchangensis* and the modern baleen whale. **(A)** Reconstruction of skull roof of *Hupehsuchus nanchangensis*, based on the new specimens, 2020-NYF-84-4 and WGSC V26007. **(B)** Skull roof of an adult minke whale from [28] - used following the attainment of appropriate copyright permission. The skull roofs are signed with 9-landmark configuration and descriptions seen in Table 1. The grey region is the paired nasals

**Fig. 3:**
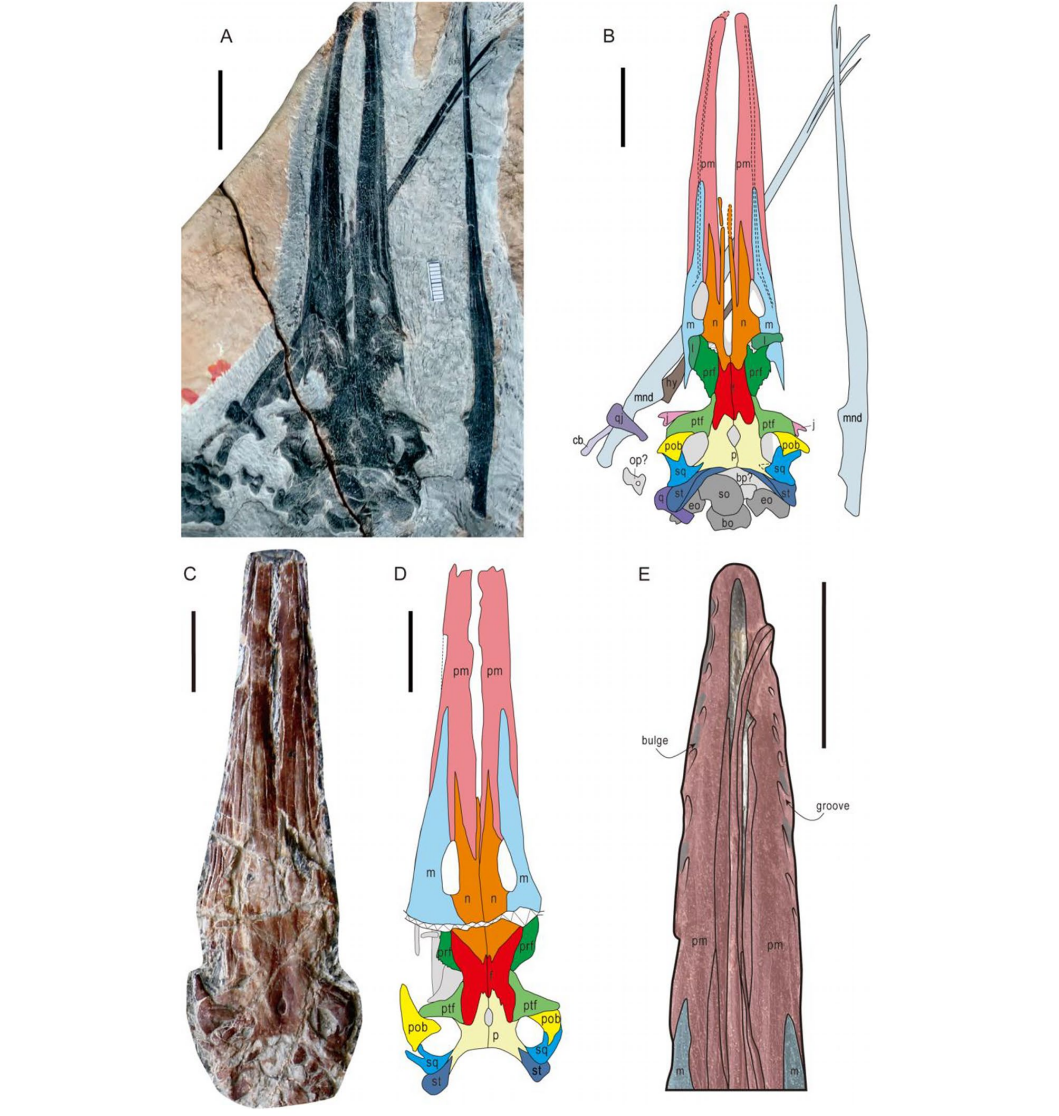
The morphological details in skulls of *Hupehsuchus nanchangensis* **A-B.** The cranial photograph and interpretative drawing of 2020-NYF-84-4 in dorsal view. Dashed lines indicate the long grooves that run along the labial margin. **C-D.** The cranial photograph and interpretative drawing of WGSC V26007 in dorsal view. Dashed line represents broken in specimen. **E.** The palatal view of the skull of referred specimen WGSC V26000, the black lines outline the groove-like depressions, and the grey regions highlight the bulges around the palatal margin **Abbreviations:bo:** basioccipital; **bp:** basisphenoid; **cb:** ceratobranchial; **eo:** exoccipital; **f:** frontal; **hy:** hyoid; **j:** jugal; **l:** lacrimal; **m:** maxilla; **mnd:** mandible; **n:** nasal; **op:** opisthotic; **p:** parietal; **pm:** premaxilla; **pob:** postorbital; **ptf:** postfrontal; **prf:** prefrontal; **q:** quadrate; **qj:** quadratojugal; **sp:** splenial; **sq:** squamosal; **so:** supraoccipital; **st:** supratemporal; **utf:** upper temporal fenestra. Scale bars = 2 cm.

We compare the hupehsuchian and cetacean and other modern aquatic predator skulls using landmark analysis. Considering the better condition in 2020-NYF-84-4 with little displacement, as opposed to the broken tip of the rostrum and compressive damage observed in WGSC V26007 (Fig. 2A–D), we selected the skull of 2020-NYF-84-4 for reconstruction (Fig. 3A) and landmark measurement. The modern sample comprises skulls of 130 amniote species (15 mysticetes, 52 odontocetes, 23 pinnipeds, 14 crocodilians, 25 birds, and one platypus, with detailed information provided in the Supplemental Information), which live in a variety of aquatic environments, including marine and riverine. We established a set of nine landmarks in the skull roof (Table 1), with reference to [27, 28]. These landmarks describe the basic outline of the skull roof, as well as the relative length and intermediate space of the rostrum (Fig. 3). Landmark placement was undertaken in software tpsUtil and tpsDig [29, 30]. All landmarks were aligned using a generalised Procrustes analysis (GPA) to remove the noise effects of size, position and rotation [31]. During the GPA, the landmarks were allowed to iteratively slide to minimise Procrustes distances between each specimen and the average shape [32]. The resulting set of aligned landmark coordinates was then subjected to principal component analysis (PCA) to examine critical components of shape variation. GPA and PCA were conducted in PAST [33]. The landmark data is provided in the see Supplemental Information for Table S2.

**Table 1:** List of landmarks used for the morphospace analyses depicted in Fig. 3, follow the reference [27]

9-landmarks in skull roof configuration	
1,7	Anteriormost point of the premaxilla
2,6	Widest point of the skull
3,5	Posteriormost point of the skull
4	End of the skull in middleline
8	Anteromedial point of nasals (end of the beak in the middle line in birds)
9	Posteromedial point of nasals (the cranio-facial hinge in the middle line in birds)

To verify the relationship between morphological convergence and feeding performance, we compiled prey size classes from previous literature [2, 3, 34]. Relative prey sizes reflect the dietary sources and are calculated by dividing the maximum length of the longest prey species by the maximum length of the predator [2]. McCurry et al. [2] divided prey size into four categories: <50%, 50%~100%, 100%~150%, and > 150%. In marine trophic strcuture, some tiny organisms, such as zooplankton, are key sources of nutrition, so this study suggests that they should be classified separately. In addition, prey items that larger than the size of the predator would be grouped into another category of large prey. Four categories of prey size were defined in this paper: tiny, small, middle, and large (see Supplemental Information for Table S1). Taxa in the tiny class specially feed on zooplankton or fishes that are far smaller than them. Predators regarded as feeding on small class prey consume items ranging from zooplanktons or benthic invertebrates to small fishes and squid, whereas those in the middle class are usually fish and squid specialists. The large class represents apex predators that prey on tetrapods.

## Results

### Skull morphology

The new specimens of *Hupehsuchus nanchangensis* are exposed in dorsal view and display a strange snout structure in which the skeleton is divided into right and left crura surrounding a narrow median space (Fig. 2A–D). The intercrural space is exceptionally long and bordered by premaxillae and nasals. In 2020-NYF-84-4, the edge of the intercrural space is smooth in natural preservation, excluding the possibility that it was broken (Fig. 2A, B). In WGSC V26007, on the other hand, there is an incomplete gap, and the premaxillae show crushed inner margins, suggesting breakage of the intercrural space after burial (Fig. 2C, D). The premaxillae are elongated and gradually widen from the tip of the snout to the end. There are long grooves inside the premaxillae that run along the labial margin, continuously entering the maxillae, as in *Eretmorhipis carrolldongi* [18] (Fig. 2A, B). The paired nasals with anterior forked processes contact each other only in their posteromedian portion because the intercrural space separates their anterior portions.

Several skull morphological characters are confirmed in the new specimens. The oval external naris is surrounded by the maxilla and nasal, and the premaxilla enters the external naris. As previously observed [35], the prefrontal contacts the postfrontal by a thin suture to prevent the frontal from entering the orbital margin. The upper temporal fenestra is surrounded by the postorbital, parietal, postfrontal and squamosal, while the supratemporal is excluded from the upper temporal fenestra (Fig. 2A–D). The jugal has a short posterior process seen as an ancestral character in Ichthyosauromorpha [36]. The mandible, complete in 2020-NYF-84-4, is extremely slender with the pronounced retroarticular process showing a trapeziform shape (Fig. 2A, B). The mandible becomes narrow from the median region to the tip. Posteriorly, the mandible has two obtuse eminences on its dorsal margin to the coronoid process, forming a low shelf. This condition is different from that of Ichthyosauriformes, but reminiscent of the coronoid process and the articular condyle present in baleen whales [26, 37]. Anteriorly, the mandible forks into two processes at the tip, probably caused by separation of the dentary and splenial. The paired mandibles enclose the lower jaw region over a large range. Besides, the mandibular symphysis in 2020-NYF-84-4 is discrete without any breaks as observed before [26]. The separate mandibles that loosely articulate with the skull resemble those of modern rorqual whales, which are efficient means to expand a large gular pouch [38]. Lateral palatal foramina, which provide an osteological correlate for inferring the presence of baleen in mysticetes, are not observed in the palate of WGSC V26000 (Fig. 2E), but the jaw margin has a series of oblique parallel shallow groove-like depressions, oriented from rostromedially to caudolaterally [26] (Fig. 2E). There are several bulges in the same orientation and between the grooves.

### Landmark morphospace analysis

**Fig. 4:**
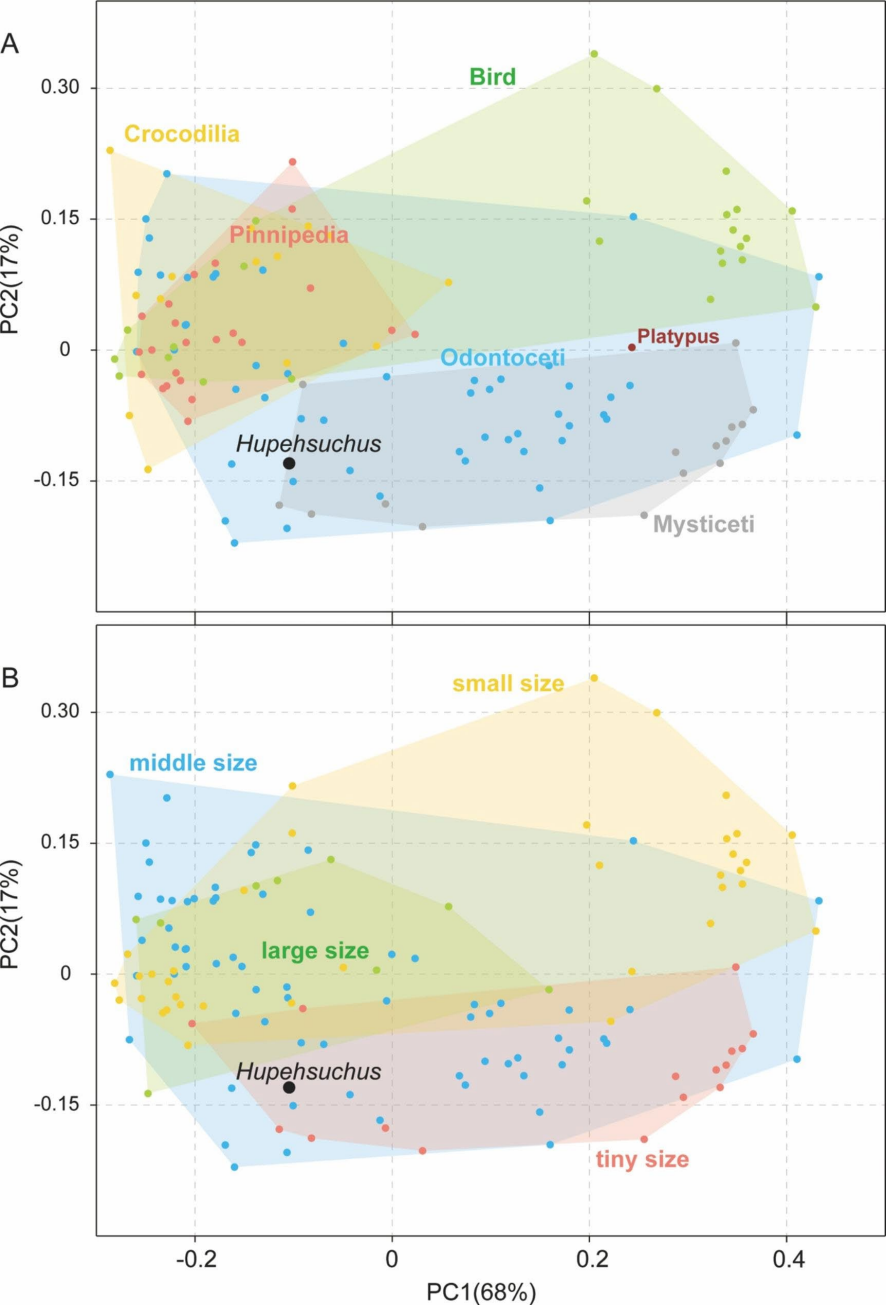
Morphospace of the skull roof in marine amniotes based on landmark analysis. **(A)** Distribution of key taxonomic groups. **(B)** Distribution of the ecomorphological guilds of predators based on prey size. The analysis includes nine landmark points, and the two-dimensional morphospace plots are based on the first two principal components, with *Hupehsuchus* placed in context of data from modern marine mammals

In the principal component analysis, most shape variation in the skull is summarised by the first two principal component axes (PCs), accounting for 67.2% and 17.5% of variation, respectively. PC1 reflects changes in relative length of the snout and PC2 highlights differences in maximal width of the skull. In the morphospace (Fig. 4A), odontocetes occupy the largest region and overlap with other groups, reflecting their high species richness and functional diversity. The second most dispersed group, the birds, nearly all have positive PC2 scores, but divide into two parts along the PC1 axis, with negative and positive scores. The mysticetes nearly all have negative PC2 scores, overlapping part of the region of odontocetes exclusively. The crocodilians and pinnipeds show high levels of overlap, located in negative PC1 regions, except for *Paleosuchus palpebrosus* and *Ommatophoca rossii*. The morphospace of the last three groups is relatively restricted, reflecting the specialisation of these groups. Birds, odontocetes and (crocodilians/ pinnipeds) all occupy non-overlapping areas of morphospace.

The point for *Hupehsuchus* is located in the morphospace where the mysticetes overlap the odontocetes, indicating that its skull shape is similar to that of modern whales (Fig. 4A). *Hupehsuchus* shares the elongated snout and posterior movement of the nasals with modern baleen whales. In addition, the intercrural space in its palate is similar to the mesorostral groove in cetaceans, separating the premaxillae. The skull of *Hupehsuchus* is more similar to that of mysticetes than odontocetes in the elongated separated rostrum, the toothless snout, and concave braincase in the midline [39]. Differing from this, odontocetes have more posterior migration of the nasals, development of a more rounded braincase, and increasing facial asymmetry [40].

The morphological convergence between *Hupehsuchus* and mysticetes is matched by association with prey size (Fig. 4B). Almost all mysticetes prey on tiny-sized zooplankton, whereas odontocetes, pinnipeds, and birds prey on small to middle-sized invertebrates, squids and fishes [34]. Odontocetes and crocodilians select prey over a wide size range, from small fish to large tetrapods, some reaching apex predatory niches, such as *Orcinus orca* and *Crocodylus porosus* [41, 42]. In the prey-size morphospace (Fig. 4b), *Hupehsuchus* is located in the region overlapped by tiny and middle-size predators, which corresponds to its edentulous snout, slender mandible with flexural rigidity and living in an environment lacking fishes and crustaceans [14].

## Discussion

### Morphological implications and comparison of the new skulls in ***Hupehsuchus***

The new skulls reveal the existence of an intercrural space in the snout of *Hupehsuchus nanchangensis*, which is morphologically convergent with the mesorostral groove in modern rorquals, supported by the morphometric analysis [43]. Besides expanding a gular pouch through flexible and elongated mandibles, *Hupehsuchus nanchangensis* increased the intercrural space in the snout to widen and enlarge the buccal cavity to adapt to filter feeding. Filter-feeding tetrapods require a large mouth to make predation efficient [44, 45]. In the evolution of mysticetes, a wider and loose rostrum is a critical adaptation for filter feeding, which occurred before the existence of baleen [46]. During development, modern baleen whales show progressive elongation of the rostrum relative to the braincase and positive allometry of the skull, which is related to their need to develop a large buccal cavity to perform filter feeding [28, 46, 47]. Similarly, the filter-feeding Cretaceous plesiosaur *Morturneria seymourensis* had a deeply arched palate with a midline keel to increasing buccal cavity volume, convergent with baleen whales, like gray whales [8, 48, 49].

The intercrural space in *Hupehsuchus nanzhangensis* is comparable with a similar structure in *Eretmorhipis carrolldongi*, a hupehsuchian with small eyes, which might have been a predator that used non-visual senses [18]. However, there are several distinct osteological differences between these two species: the intercrural space in *Hupehsuchus* is exceptionally slender, surrounded by the premaxillae and the nasals, whereas in *Eretmorhipis* it is oval and only surrounded by the premaxillae; there is an isolated bone in the intercrural space of *Eretmorhipis*, whereas the same bone is not found in *Hupehsuchus*; the snout length relative to the skull of *Hupehsuchus* is longer than in *Eretmorhipis*. Further, *Eretmorhipis* was a slow manoeuvring swimmer with a rigid body and tail coupled with large fan-shaped propulsive flippers, and small-sized eyes, suggesting non-visual prey detection [18]. *Hupehsuchus* had larger eyes and a slenderer snout than *Eretmorhipis*. The anatomy of both taxa suggests that *Hupehsuchus* was a better swimmer than *Eretmorhipis*, which would imply different feeding strategies.

Some other Mesozoic marine reptiles show a similar space or foramen in the midline of the snout region, but this might have had a variety of functions. The Middle Triassic *Atopodentatus unicus*, with its pronounced hammerhead-shaped skull, has paired separated premaxillae with a slender rhombus-shaped space [11]. Its heterodont teeth, the chisel-shaped teeth in the straight anterior edge of the jaws, and the needle-shaped jaw ramus suggest that this unusual marine reptile was a seaweed grazer, the oldest record of herbivory in a marine reptile [11]. The edentulous Late Triassic ichthyosaur *Shastasaurus liangae* (= *Guanlingsaurus liangae*) shows a very large internasal foramen in the skull roof [50, 51]. The feeding mode of this ichthyosaur has been debated; perhaps it was a suction feeder based on its short toothless snout, or perhaps the slender hyobranchial bone excludes the affinity with suction feeding, suggesting it was a ram feeder [50, 52, 53]. Further, in the Early Jurassic ichthyosaurs *Ichthyosaurus communis* and *Leptonectes tenuirostris*, the foramen in the skull roof midline moves posteriorly to the internasal and interfrontal region [54, 55].

### The model of filter feeding in ***Hupehsuchus***

Baleen is made from keratin, forming a soft and tough fibrous curtain dangling from the upper jaw in baleen whales, and used to filter engulfed water in the mouth and trap prey [56]. The origin of baleen in stem mysticetes is contentious and researchers suggested several interpretations of the transition from raptorial feeding with teeth as in stem mysticetes to baleen-assisted filter feeding as in modern mysticetes [57–60]. However, the best explanation supported by current evidence on this transition is that the stem mysticetes passed through an intermediate stage with both teeth and baleen before complete loss of their teeth and becoming modern filter feeders with baleen [39, 57]. The lateral palate foramina in the stem mysticetes, which are homologous with neurovascular structures that nourish and innervate the baleen apparatus in extant mysticetes, are associated with the presence of baleen in this hypothesis [61]. In *Hupehsuchus*, the grooves and bulges around the labial margins are reminiscent of the lateral palate foramina in mysticetes, suggesting the existence of soft tissues like baleen during the whole feeding process, and these presumably played an important role in filter feeding. In life, these grooves may have borne soft tissues for filter feeding, which replaced the position of the dental alveoli, similar to grey whales [62]. Although we cannot identify soft tissues in the fossils, these uneven structures would have been useful to strain the water expelled from the mouth cavity, completing the filtration. Therefore, we argue that the cranial structure of *Hupehsuchus* is convergent with modern baleen whales on the basis of three characters: the intercrural space in the snout, the slender and unfused lower jaws, and the grooves left by soft tissues around the palatal margins. Perhaps the diet of *Hupehsuchus* resembled that of modern mysticetes, which depend on the supply of zooplankton, such as shrimp-like arthropods. The laminated limestone in the NYF indicates sufficient zooplankton for *Hupehsuchus* [13, 14].

Although the modern baleen whales are all large filter feeders, they feed quite differently in terms of strategy and food preference [34, 49]. The balaenopterid whales, also known as rorquals, employ a lunge filter feeding style in which they swim rapidly at a prey patch while opening their mouth to gulp the mixture of water and prey, then filter the water through the baleen plates and swallow the retained prey [44, 63, 64]. Rorqual whales have specialised anatomy and feeding performance to support their lunge feeding strategy to capture fish shoals and plankton [38, 65, 66]. The balaenid whales, including bowhead and right whales, employ a skim filter feeding style in which they capture plankton from the water by swimming slowly with their mouth open [67]. In another filtering mode, the grey whale (*Eschrichtius robustus*) feeds mainly on benthic invertebrates that it ingests by swimming along the seabed on one side, using lateral suction feeding to take in sediment plus prey [5, 68, 69].

Previously, Motani et al. [26] suggested that *Hupehsuchus* was a lunge feeder like pelicans or rorquals, based mainly on its slender and flexible mandible, and its palatal structure which probably supported soft tissues as strainer. The cranial structure in this study reveals that *Hupehsuchus* is more like baleen whales than pelicans and employed filter feeding. Considering its paddle-like limbs and high dorsal neural spines, *Hupehsuchus* was thought to have advantages for acceleration and manoeuvring, as in intermittent lunge filter feeders like rorquals [26]. But the rigid trunk without intercostal space and three layers of dorsal dermal ossicles in an imbricate arrangement limited the aquatic locomotion by lateral axial undulation as anguilliform swimming which is common in early Ichthyosauromorpha [35, 70]. The pachyostotic ribs in *Hupehsuchus* indicate the function of buoyancy control and swimming in shallow-water inhabitants [71]. Thus, *Hupehsuchus* would have employed continuous ram filter feeding as in bowhead and right whales, rather than lunge filter feeding as in rorqual whales [72]. The NYF environment lacking fishes also indicates that *Hupehsuchus* could have fed only on zooplankton, unlike rorquals that feed on fishes [13, 14, 18]. The hyoid bone in *Hupehsuchus* is not strong enough to support suction feeding as in grey whales [26, 53, 73]. *Hupehsuchus* would have continuously filter fed at slow swimming speeds, from dense patches of plankton at the surface or shallow water column. The mandible with a well-developed retroarticular process and rostral bones that accommodate the intercrural space improve the functional advantage of the volume of the oral cavity, which is efficient for filter feeding.

### The evolution and implication of filter feeding in ***Hupehsuchus***

**Fig. 5:**
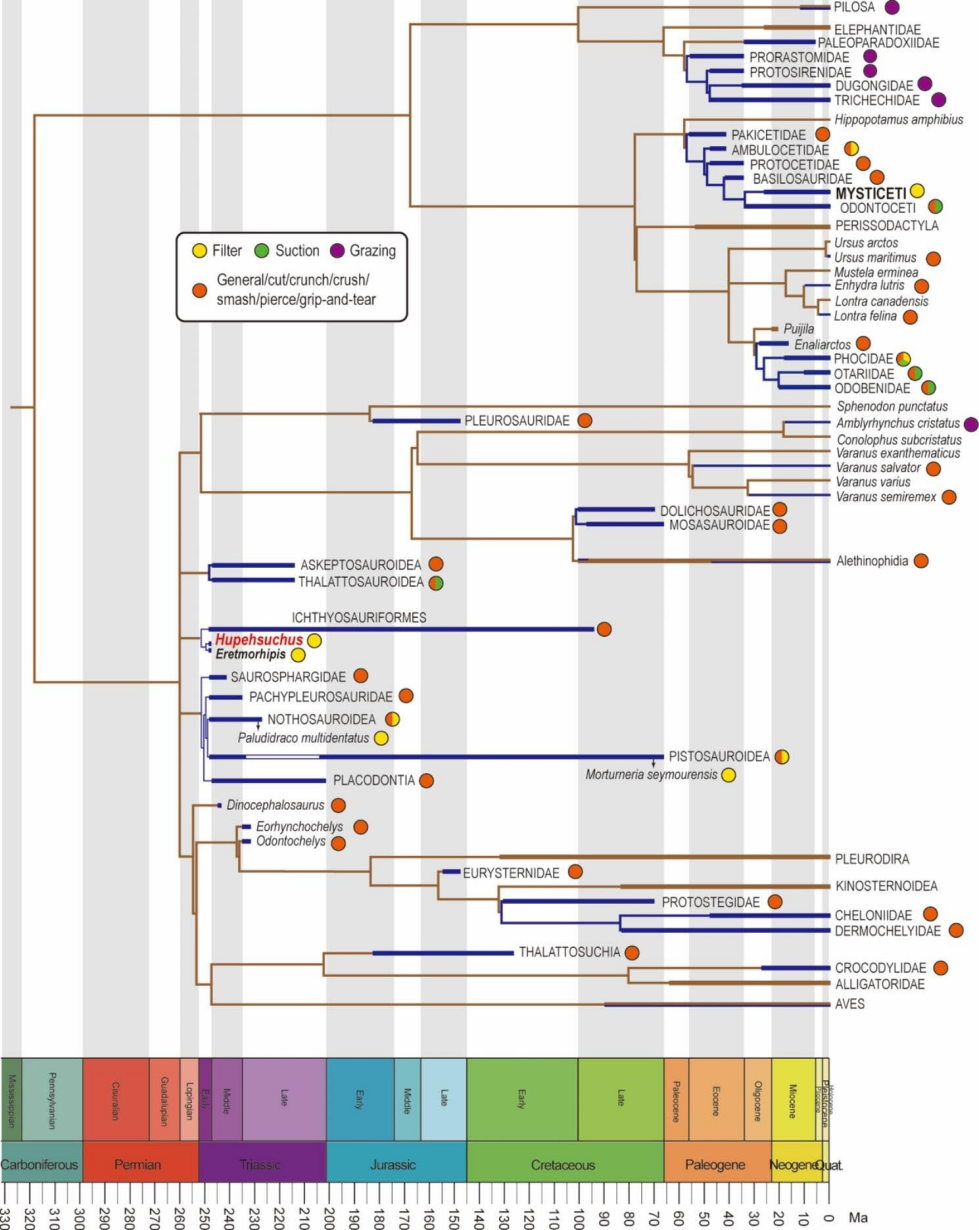
Phylogenetic tree of major groups of marine tetrapods with selected terrestrial sister taxa modified from [81], following the attainment of appropriate copyright permission. The groups and species are marked with their feeding guilds. The blue branches indicate marine-adapted tetrapods and brown branches represent their terrestrial sister taxa. The classifications of feeding guilds are from [34] for mammals, [78] and [81] for reptiles

Hupehsuchians and mysticetes specialize in a filter-feeding strategy, but there is a major difference in the speed with which this unusual feeding mode evolved in the two clades. Whereas whales evolved 15 Myr after the end-Cretaceous mass extinction (66 Ma), and filter-feeding adaptations in mysticetes long after that (34 Ma), marine reptiles diversified extraordinarily fast in the Early and Middle Triassic [74–77], acquiring a broad array of adaptations within as little as 5 Myr (Fig. 5). The diversity of feeding guilds of Triassic marine ecosystems is comparable to that in the modern marine environment [78]. The cranial morphology of Mesozoic marine reptiles reflects their feeding modes and is usually divided into brevirostrine and longirostrine types, reflecting short and long snouts, respectively. Brevirostrine marine reptiles included suction feeders which created sub-ambient pressure in the mouth to capture prey, such as most eosauropterygians and thalattosaurs [79, 80]. Longirostrine marine reptiles, including almost all ichthyosaurs, are generally regarded as ram feeders whose acceleration and movement are used in prey capture [52, 53]. We now add to this diversity in Early Triassic feeding guilds the first confirmation of filter feeding in *Hupehsuchus*.

Secondary aquatic adaptation by Mesozoic reptiles and Cenozoic mammals provides many classic examples of convergent evolution, explained as adaptations to similar ecological niches [1, 81]. Constraints on locomotion in the aquatic environment may have enhanced the repeated convergences in body plan. Many marine reptiles and mammals evolved streamlined bodies and efficient lift-based swimming, especially using propulsion from the dorsal fin in ichthyosaurs and modern dolphins [82]. Further, cranial and tooth morphology can reflect trophic convergence related to food resources, feeding strategies, and prey levels. Driven by filtering adaptations, *Hupehsuchus* and baleen whales share convergences which are concentrated in their cranial morphology, such as the edentulous snout, the divided upper jaw and the mandible with a pronounced retroarticular process.

*Hupehsuchus* lived in the Early Triassic, and it was part of the rapid biotic recovery of complex marine ecosystems after the end-Permian mass extinction [17, 83]. The NYF in which it occurs is characterized by its restrictive lagoonal paleoenvironment, high reptile diversity and absence of fishes and invertebrates [13, 14]. Perhaps *Hupehsuchus* showed innovative skull morphology to adapt to filter feeding as a result of competition from other predatory marine reptiles such as ichthyosaurs and eosauropterygians, and as a means to benefit from a food resource that was not otherwise fully exploited.

## Conclusion

The shape of the skull roof and snout of *Hupehsuchus nanchangensis* is highly convergent with modern baleen whales. The intermediate space in the snout and the unfused premaxillae of *Hupehsuchus nanchangensis* enabled it to enlarge the buccal cavity, a key requirement for an oral filter-feeding tetrapod. The remodelling of the snout, with the flexible slender mandible, promoted its filter-feeding efficiency. *Hupehsuchus* was more probably a continuous ram filter feeder like extant bowhead and right whales, based on its low swimming speeds revealed by the rigid trunk. We found that the specialization is associated with prey size and contributes to the dominance of *Hupehsuchus* in the Nanzhang-Yuan’an Fauna. What is remarkable is that, whereas it took some 30 Myr for whales to evolve filter-feeding adaptations, this was achieved in less than 5 Myr by *Hupehsuchus* in the Early Triassic.

## Electronic supplementary material

Below is the link to the electronic supplementary material.

Supplementary Material 1

## Data Availability

All data generated or analysed during this study are included in this published article and its online supplementary information file. The specimens of *Hupehsuchus nanchangensis*, WGSC V26007 and 2020-NYF-84-4, are respectively housed at Wuhan Centre of China Geological Survey and Yuan’an Geology Museum.
